# Preoperative routine measurement of NT-proBNP predicts postoperative morbidity after non-cardiac surgery with intermediate or high surgical risk: an observational study

**DOI:** 10.1186/s12871-024-02488-8

**Published:** 2024-03-23

**Authors:** Götz Schmidt, Nora Frieling, Emmanuel Schneck, Marit Habicher, Christian Koch, Kerstin Rubarth, Felix Balzer, Birgit Aßmus, Michael Sander

**Affiliations:** 1 Department of Anaesthesiology, Operative Intensive Care Medicine and Pain Therapy, Justus Liebig University Giessen, Rudolf-Buchheim-Strasse 7, 35392 Giessen, Germany; 2 Institute of Medical Informatics, Charité – Universitätsmedizin Berlin, corporate member of Freie Universität Berlin and Humboldt- Universität zu Berlin, Charitéplatz 1, 10117 Berlin, Germany; 3 Department of Cardiology and Angiology, Justus Liebig University of Giessen, Klinikstrasse 33, 35392 Giessen, Germany

**Keywords:** Perioperative, Brain natriuretic peptide, Infection, Rehospitalisation, Acute kidney injury, Acute decompensated heart failure

## Abstract

**Background:**

Chronic heart failure (HF) is a common clinical condition associated with adverse outcomes in elderly patients undergoing non-cardiac surgery. This study aimed to estimate a clinically applicable NT-proBNP cut-off that predicts postoperative 30-day morbidity in a non-cardiac surgical cohort.

**Methods:**

One hundred ninety-nine consecutive patients older than 65 years undergoing elective non-cardiac surgery with intermediate or high surgical risk were analysed. Preoperative NT-proBNP was measured, and clinical events were assessed up to postoperative day 30. The primary endpoint was the composite morbidity endpoint (CME) consisting of rehospitalisation, acute decompensated heart failure (ADHF), acute kidney injury (AKI), and infection at postoperative day 30. Secondary endpoints included perioperative fluid balance and incidence, duration, and severity of perioperative hypotension.

**Results:**

NT-proBNP of 443 pg/ml had the highest accuracy in predicting the composite endpoint; a clinical cut-off of 450 pg/ml was implemented to compare clinical endpoints. Although 35.2% of patients had NT-proBNP above the threshold, only 10.6% had a known history of HF. The primary endpoint was the composite morbidity endpoint (CME) consisting of rehospitalisation, acute decompensated heart failure (ADHF), acute kidney injury (AKI), and infection. Event rates were significantly increased in patients with NT-proBNP > 450 pg/ml (70.7% vs. 32.4%, *p* < 0.001), which was due to the incidence of cardiac rehospitalisation (4.4% vs. 0%, *p* = 0.018), ADHF (20.1% vs. 4.0%, *p* < 0.001), AKI (39.8% vs. 8.3%, *p* < 0.001), and infection (46.3% vs. 24.4%, *p* < 0.01). Perioperative fluid balance and perioperative hypotension were comparable between groups. Preoperative NT-proBNP > 450 pg/ml was an independent predictor of the CME in a multivariable Cox regression model (hazard ratio 2.92 [1.72–4.94]).

**Conclusions:**

Patients with NT-proBNP > 450 pg/ml exhibited profoundly increased postoperative morbidity. Further studies should focus on interdisciplinary approaches to improve outcomes through integrated interventions in the perioperative period.

**Trial registration:**

German Clinical Trials Register: DRKS00027871, 17/01/2022

**Supplementary Information:**

The online version contains supplementary material available at 10.1186/s12871-024-02488-8.

## Background

Chronic heart failure (HF) is associated with reduced quality of life and increased morbidity and mortality, and its prevalence is increasing, especially in ageing and growing populations [1–3]. While the overall prevalence of chronic HF is estimated to be 1 − 2%, more than 10% of patients older than 65 years suffer from chronic HF [4]. Patients with HF undergoing non-cardiac surgery are at increased risk of haemodynamic alterations during general anaesthesia in addition to the risks associated with surgical trauma [5]. Current European Society of Cardiology (ESC) guidelines on cardiovascular assessment and the management of patients undergoing non-cardiac surgery recommend that perioperative risk should be assessed by evaluating patient-related and surgery-related risk factors. The 30-day risk for cardiovascular death, stroke, and myocardial infarction stratified solely by the type of surgery is between 1% and 5% for intermediate-risk and > 5% for high-risk surgeries, regardless of the patient’s history [6]. These findings not only indicate the need for careful management of elderly patients with known HF requiring major non-cardiac surgery but also the necessity to detect patients with undiagnosed HF who might be at risk of increased peri- and postoperative morbidity. Although elderly patients undergoing major surgery should be carefully examined for HF according to the current guidelines, diagnosis of HF by history and physical examination alone may be challenging during the preoperative evaluation [6]. Measurement of cardiac biomarkers can be used to risk-stratify patients undergoing surgery. Plasma brain natriuretic peptide (BNP) and its precursor, N-terminal prohormone of BNP (NT-proBNP), are relevant prognostic markers that can predict mortality, stroke, and cardiovascular adverse events in asymptomatic persons [7]. In the new 2022 ESC guidelines on cardiovascular assessment and management in patients undergoing non-cardiac surgery, the measurement of NT-proBNP has a Class IIa recommendation for application in patients with known cardiovascular disease, cardiovascular risk factors, or symptoms suggestive of cardiovascular disease undergoing non-cardiac surgery with intermediate or high surgical risk [6]. However, while routine NT-proBNP measurements are still not recommended (Class III recommendation), preoperative measurement of natriuretic peptides has been shown to effectively predict major cardiac adverse events after major non-cardiac surgery in different patient populations [8–11]. Most studies have focused on cardiac mortality and postoperative myocardial injury or infarction, where the incremental predictive value of NT-proBNP in predicting mortality and myocardial injury was observed [11]. .

Therefore, different NT-proBNP cut-offs covering a wide range of values, from 200 pg/ml up to 1,500 pg/ml, for risk stratification regarding the risk of death and myocardial injury in patients undergoing major non-cardiac surgery have been proposed [11–13]. A multicentric observational study recently demonstrated a 2.5% rate of postoperative acute decompensated HF (ADHF) following non-cardiac surgery, and 44% of patients with postoperative ADHF died within one year after surgery [14]. Interestingly, 51% of patients with postoperative ADHF presented with de novo HF and had no prior history of HF. Because preoperative NT-proBNP was only available in 24% of the cohort, the findings underlined the need for further studies evaluating the predictivity of NT-proBNP concerning the postoperative morbidity, while further perioperative measures, such as blood loss, fluid balance, use of diuretics, and perioperative haemodynamics, should also be considered [15]. This study aimed to evaluate postoperative morbidity within 30 PODs, including rehospitalisation, ADHF, acute kidney injury (AKI), and infection. Additionally, the study should estimate a cut-off value for NT-proBNP in the routine preoperative assessment of patients older than 65 years undergoing elective intermediate- or high-risk non-cardiac surgery to predict these outcomes. Furthermore, the benefit of including NT-proBNP in the prediction of postoperative morbidity in the context of other preoperatively available clinical and laboratory parameters was analysed.

## Methods

### Study design

This prospective, single-centre cohort study was approved by the local ethics committee of the medical faculty of the Justus-Liebig-University, Giessen, Germany (AZ 263/21), and was performed in compliance with the Declaration of Helsinki. According to the observational nature of the entire study and subsequent data anonymisation, written informed consent to participate was waived. This study was registered with the German Clinical Trials Register (DRKS00027871; date of registration: 17/01/2022). Consecutive patients older than 65 years undergoing intracranial, thoracic, head and neck, trauma and orthopaedic, or abdominal non-cardiac surgery, including visceral, urological, and gynaecological operations with intermediate or high surgical risk under general anaesthesia received routine preoperative NT-proBNP measurement. Prior to the presurgical anaesthetist visit, NT-proBNP was measured from venous blood using a point-of-care immunoassay (proBNP+, cobas h 232, Roche Holding AG, Basel, Switzerland). Anaesthetic management and intra- and postoperative treatments were provided according to local institutional standards in compliance with the current guidelines.

### Data acquisition

Preoperative data were collected during the routine presurgical visit at the anaesthesia outpatient clinic. Baseline characteristics included age, sex, body mass index, cardiovascular risk factors, revised cardiac risk index, history of chronic HF or myocardial infarction, and relevant comorbidities, such as peripheral artery disease, carotid artery stenosis, chronic obstructive pulmonary disease, pulmonary hypertension, stroke, and chronic kidney disease. Surgical data involved the type and speciality of surgery and the surgical risk classification stratified by “intermediate risk” or “high risk”. If preoperative echocardiographic findings were available, the findings were reported, and perioperative fluid balance, including blood and fluid losses and the amount of fluid and blood product administered, was assessed. Further perioperative measurements included pre- and postoperative haemoglobin (Hb), serum creatinine, estimated glomerular filtration rate (eGFR), the amount of catecholamine administered, administrative procedural data, and intraoperative haemodynamics. Anaemia was defined according to the World Health Organization as Hb levels < 13 g/dL in men and < 12 g/dL in women, respectively [16]. Perioperative hypotension was assessed during anaesthesia and was defined as mean arterial pressure < 65 mmHg. Blood pressure data were recorded automatically every 3 min in the digital anaesthetic protocol, and the incidence, duration (minutes), and severity of hypotension expressed as area under the curve (mmHg × min) were calculated. To assess postoperative events, clinical records were screened daily during the hospital stay, and clinical follow-up was conducted at POD 30. When unplanned rehospitalisation occurred, clinical records were requested and subsequently reviewed.

### Endpoints

The primary endpoint was the composite morbidity endpoint (CME) consisting of the incidence of rehospitalisation, ADHF, AKI, and any suspected or proven bacterial infection requiring treatment at POD 30. AKI was defined according to the Kidney Disease: Improving Global Outcomes criteria [17]. ADHF was defined as the onset or worsening of shortness of breath and signs of congestion, including peripheral oedema, moist rales, and radiological signs of congestion or pleural effusion requiring treatment [18]. Secondary endpoints included the individual components of the composite endpoint, myocardial infarction, wound healing disturbances, and mortality at POD 30. During the hospital stay, the administration and cumulative doses of intravenous diuretics (furosemide equivalent in mg per day) were recorded, and the length of intensive care unit and hospital stay were calculated.

### Statistical analysis

As no previous clinical data regarding the statistical distribution of preoperative NT-proBNP values were available in our study cohort, no formal power analysis was performed. To obtain a sufficient group of patients, a sample size of 200 pilot patients undergoing routine preoperative NT-proBNP measurement was considered sufficient to assess the clinical endpoint measures. Because different cut-offs for NT-proBNP are reported for composite endpoints consisting of mortality and myocardial injury or infarction, there is an overall lack of consensus regarding the cut-off for elevated NT-proBNP in different settings and age groups. Therefore, the area under the receiver operating characteristic (ROC) was initially calculated to determine the discriminatory power for CME using NT-proBNP [19, 20]. The optimal discriminatory level of NT-proBNP was identified according to Youden’s index. The events of patients with NT-proBNP above the implemented cut-off were compared to their respective complementary groups. Event rates were calculated using the Kaplan-Meier method, and statistical differences were assessed using the log-rank test. Categorial variables are presented as numbers and percentages, and the Chi-squared test or Fisher’s exact test was used to evaluate differences between groups. Continuous variables are presented as median and interquartile range and were compared using the Mann-Whitney-Wilcoxon test. Multivariable Cox regression analysis was performed for the CME to identify independent predictors and differentiate the association of the preoperative NT-proBNP threshold in comparison to relevant clinical parameters and the revised cardiac risk index. Two-tailed values of *p* < 0.05 were considered statistically significant. Statistical analyses were performed using IBM SPSS Statistics, version 28.0.0.1 (IBM Corp., Armonk, NY, USA) and R 4.2.3 (www.r-project.org).

## Results

Two hundred patients underwent preoperative NT-proBNP measurement prior to non-cardiac surgery with intermediate or high surgical risk and general anaesthesia between January and March 2022. Because one surgery was cancelled for surgical reasons, 199 patients were included in the final analysis.

### NT-proBNP cut-off

**Fig. 1:**
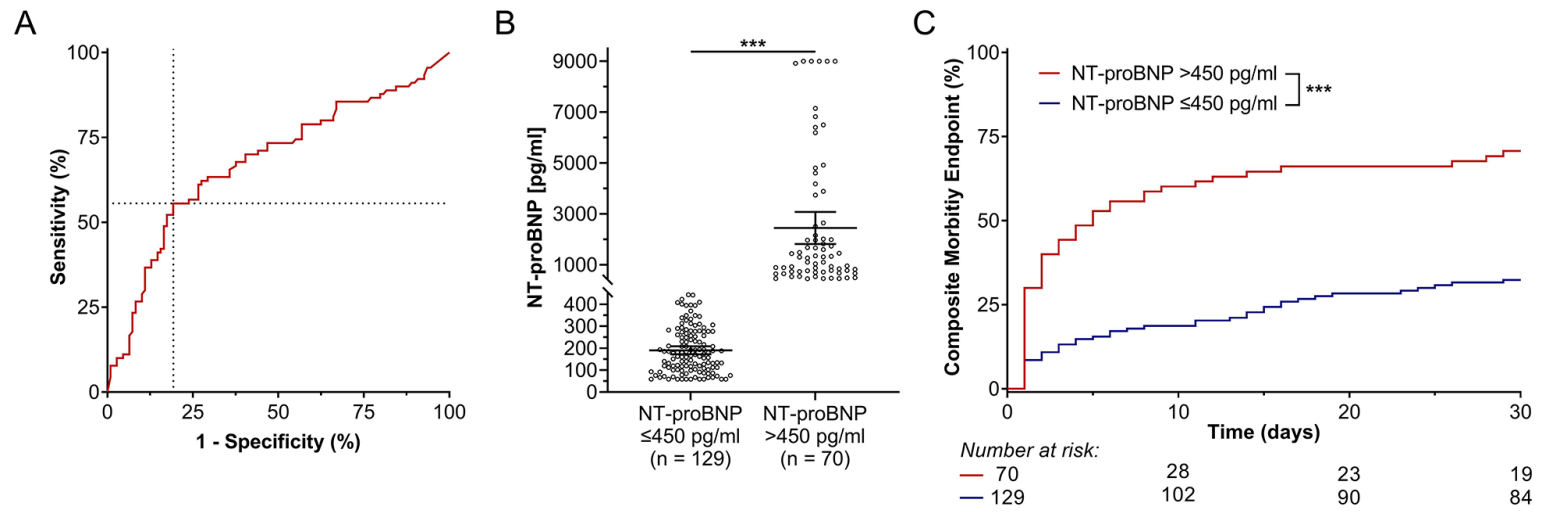
Preoperative NT-proBNP and primary endpoint. **A** NT-proBNP of 443 pg/ml had the highest accuracy in predicting the composite endpoint (area under the receiver operating characteristic curve 0.679, *p* < 0.001, Youden’s index 0.363, sensitivity 55.6%, specificity 80.7%, dotted line). **B** Distribution of NT-proBNP in both analysed groups. **C** Kaplan-Meier estimates revealed adverse 30-day outcomes in patients with preoperative NT-proBNP > 450 pg/ml (composite morbidity endpoint consisting of rehospitalisation, acute decompensated heart failure, acute kidney injury, and any bacterial infection). ***p < 0.001

The ROC curve indicated that NT-proBNP > 443 pg/ml had the strongest accuracy in predicting the composite endpoint (area under the ROC 0.679 [0.602–0.756], *p* < 0.001, Youden’s index 0.363, sensitivity 55.6%, specificity 80.7%, Fig. 1A). Because a cut-off > 450 pg/ml had comparable predictivity in our cohort (Youden’s index 0.352, sensitivity 54.4%, specificity 80.7%), we chose this cut-off to guarantee adequate clinical implementation as the number is easier to remember. In total, 129 (64.8%) patients had NT-proBNP ≤ 450 pg/ml and 70 (35.2%) patients had elevated NT-proBNP > 450 pg/ml. A graphical representation of the NT-proBNP distribution in both groups is shown in Fig. 1B. Median follow-up time was 30 (30–30) days in both groups, and follow-up was completed in 194 (97.5%) patients.

### Baseline characteristics

Table 1 presents the characteristics, laboratory parameters, echocardiographic findings, and surgery types of the overall population and stratified by preoperative NT-proBNP ≤ 450 pg/ml or > 450 pg/ml. Several baseline characteristics differed significantly between the two groups. Patients with NT-proBNP > 450 pg/ml were older and had higher serum creatinine, lower eGFR, and lower pre- and postoperative Hb than patients with NT-proBNP ≤ 450 pg/ml. Cardiovascular risk factors were more common in the NT-proBNP > 450 pg/ml group, which resulted in a higher rate of American Society of Anesthesiologists (ASA) classification III and functional capacity of < 4 metabolic equivalents of task in this group. Preoperative echocardiography was more frequently scheduled in patients with elevated NT-proBNP, although preoperative echocardiography was performed in only 26.6% of all cases. Left ventricular ejection fraction was comparable among groups; however, systolic pulmonary artery pressure, maximal tricuspid regurgitation velocity, E/e’ ratio and left atrial volume index differed significantly between the two groups. Patients with elevated NT-proBNP had a significant higher rate of second-to-third-grade mitral valve regurgitation (*p* = 0.011), while aortic and tricuspid valve findings were comparable between the groups.

**Table 1:** Patient characteristics

Characteristics	Total (*n* = 199)	NT-proBNP ≤ 450 pg/ml (*n* = 129)	NT-proBNP > 450 pg/ml (*n* = 70)	*p*
Patient characteristics				
Male - no. (%)	101 (50.8)	63 (48.8)	38 (54.3)	0.463
Median age [IQR] - years	74 [69–81]	72 [68–77]	80 [74–83]	**< 0.001**
Mean Body-Mass-Index [IQR] - kg/m^2^	26.3 [23.5–29.4]	26.89 [23.9–29.5]	25.3 [22.8–29.3]	0.255
Pre-existing conditions				
Arterial hypertension - no. (%)	155 (77.9)	91 (70.5)	64 (91.4)	**0.001**
Hyperlipidaemia - no. (%)	51 (25.6)	25 (19.4)	26 (37.1)	**0.010**
Nicotine abuse - no. (%)	64 (32.2)	45 (34.9)	19 (27.1)	0.338
Nicotine abuse continued - no. (%)	28 (14.1)	20 (15.5)	8 (11.4)	0.565
Diabetes mellitus - no. (%)	63 (31.7)	36 (27.9)	27 (38.6)	0.166
Peripheral vessel disease - no. (%)	19 (9.5)	9 (7.0)	10 (14.3)	0.155
Prior myocardial infarction - no. (%)	21 (10.6)	10 (7.8)	11 (15.7)	0.133
Chronic obstructive pulmonary disease - no. (%)	27 (13.6)	15 (11.6)	12 (17.1)	0.385
Pulmonary hypertension - no. (%)	4 (2.0)	0 (0.0)	4 (5.7)	**0.014**
Carotid artery stenosis - no. (%)	10 (5.0)	6 (4.7)	4 (5.7)	0.743
Chronic kidney disease - no. (%)	131 (65.8)	79 (61.2)	52 (74.3)	0.090
Stage 1 - no. (%)	68 (34.2)	50 (38.8)	18 (25.7)	0.090
Stage 2 - no. (%)	79 (39.7)	61 (47.3)	18 (25.7)	**0.005**
Stage 3 - no. (%)	37 (18.6)	17 (13.2)	20 (28.6)	**0.013**
Stage 4 - no. (%)	11 (5.5)	1 (0.8)	10 (14.3)	**< 0.001**
Stage 5 - no. (%)	4 (2.0)	0 (0.0)	4 (5.7)	**0.014**
Revised cardiac risk index [IQR] - no.	1 [0–2]	1 [0–1]	1 [1, 2]	**< 0.001**
Intraperitoneal or intrathoracic	77 (38.7)	54 (41.9)	23 (32.9)	0.274
Coronary artery disease - no. (%)	51 (25.6)	22 (17.1)	29 (25.6)	**< 0.001**
Chronic heart failure - no. (%)	21 (10.6)	4 (3.1)	17 (24.3)	**< 0.001**
HFpEF - no. (%)	15 (7.5)	3 (2.3)	12 (17.1)	**< 0.001**
HFmrEF - no. (%)	4 (2.0)	1 (0.8)	3 (4.3)	0.126
HFrEF - no. (%)	2 (1.0)	0 (0.0)	2 (2.9)	0.123
Prior stroke/TIA - no. (%)	21 (10.6)	10 (7.8)	11 (15.7)	0.133
Insulin-dependent diabetes mellitus - no. (%)	23 (11.6)	10 (7.8)	13 (11.6)	**0.041**
Serum creatine > 2 mg/dl - no (%)	13 (6.5)	1 (0.8)	12 (17.1)	**< 0.001**
ASA class				
ASA 1 - no. (%)	4 (2.0)	4 (3.1)	0 (0.0)	0.300
ASA 2 - no. (%)	67 (38.2)	59 (45.7)	17 (4.3)	**0.005**
ASA 3 - no. (%)	116 (58.3)	65 (50.4)	51 (72.9)	**0.004**
ASA 4 - no. (%)	3 (1.5)	1 (0.8)	2 (2.9)	0.283
Metabolic equivalent of task				
< 4 MET - no. (%)	31 (15.6)	10 (7.8)	21 (30.0)	**< 0.001**
Laboratory parameters				
NT-proBNP [IQR] - pg/ml	277 [134–779]	173 [103–274]	1309 [730–2918]	**< 0.001**
eGFR [IQR] - ml/min	85 [58–102]	88 [69–103]	64 [43–91]	**< 0.001**
Serum creatinine [IQR] - mg/dl	0.9 [0.7–1.1]	0.8 [0.7–1.0]	1.0 [0.8–1.4]	**< 0.001**
Preoperative haemoglobin [IQR] - g/dl	12.8 [11.3–14.2]	13.2 [12.2–14.4]	11.5 [10.1–13.6]	**< 0.001**
Postoperative haemoglobin [IQR] - g/dl	11.0 [9.8–12.5]	11.6 [10.5–12.7]	10.0 [9.3–11.6]	**< 0.001**
Echocardiography				
preoperative echocardiography - no. (%)	53 (26.6)	27 (20.9)	26 (37.1)	**0.021**
Ejection fraction				
≥ 50% - no. (%)	40 (93.0)^a^	20 (95.2)^b^	20 (90.9)^c^	1.0
41–49% - no. (%)	0 (0.0)^a^	0 (0.0)^b^	0 (0.0)^c^	1.0
≤ 40% - no. (%)	3 (7.0)^a^	1 (4.8)^b^	2 (9.1)^c^	1.0
TAPSE [IQR] - mm	23 [20-25]^d^	23 [21-25]^e^	23 [19-25]	0.250
PAP_sys_ [IQR] - mmHg	2 [22-34]^g^	23 [21-28]^h^	32 [26–39]^i^	**0.006**
TR-V_max_ [IQR] - m/s	2.6 [2.4–2.9]^j^	2.4 [2.3–2.7]^h^	2.8 [2.5–3.0]^k^	**0.010**
E/A ratio [IQR] - no.	0.8 [0.7–1.1]^m^	0.7 [0.7–1.0]^c^	0.8 [0.7–1.3]^h^	0.260
E/e’ ratio [IQR] - no.	10.6 [8.8–13.7]^n^	9.8 [8.1–11.0]^o^	11.1 [9.9–16.4]^p^	**0.029**
LA volume index [IQR] - ml/m^2^	36.7 [27.4–48.1]^m^	30.2 [26.5–43.5]^o^	47.3 [36.6–54.8]^q^	**0.017**
Aortic valve stenosis II-III° - no. (%)	1 (1.9)	1 (3.7)	0 (0.0)	1.0
Aortic valve regurgitation II-III° - no. (%)	2 (3.8)	0 (0.0)	2 (7.7)	0.236
Mitral valve regurgitation II-III° - no. (%)	9 (17.0)	1 (3.7)	8 (30.8)	**0.011**
Tricuspid valve regurgitation II-III° - no. (%)	12 (22.6)	3 (11.1)	9 (34.6)	0.086
Surgical risk				
intermediate risk - no (%)	181 (91.0)	117 (90.7)	64 (91.4)	1.000
high risk - no (%)	18 (9.0)	12 (9.3)	6 (8.6)	1.000
Surgery types				
Abdominal, thoracic - no. (%)	78 (39.2)	55 (42.6)	23 (32.9)	0.231
Neurological, maxillofacial, ear-nose-throat - no. (%)	52 (26.1)	38 (29.5)	14 (20.0)	0.200
Trauma, orthopaedic - no. (%)	69 (34.7)	36 (27.9)	33 (47.1)	**0.010**

Legend: a: *n* = 43; b: *n* = 21; c: *n* = 22; d: *n* = 49; e: *n* = 24; f: *n* = 25; g: *n* = 31; h: *n* = 13; i: *n* = 18; j: *n* = 30; k: *n* = 17; m: *n* = 35; n: *n* = 34; o: *n* = 19; p: *n* = 15; q: *n* = 16. Abbreviations: HF = heart failure, pEF = preserved ejection fraction, mrEF = mildly reduced ejection fraction, rEF = reduced ejection fraction, TIA = transient ischaemic attack, ASA = American Society of Anesthesiologists, MET = metabolic equivalent of task, eGFR = estimated glomerular filtration rate, TAPSE = tricuspid annular plane systolic excursion, PAP = pulmonary artery pressure, TR = tricuspid regurgitation, LA = left atrium

### Procedural details

Table 2 indicates the procedural details, fluid balance, catecholamine therapy, and incidence and severity of perioperative hypotension among the patients. The incidence and severity of perioperative hypotension were comparable between the groups; however, the cumulative amount of noradrenaline administered was significantly higher among patients with NT-proBNP > 450 pg/ml.

**Table 2:** Procedural details, fluid balance, catecholamine therapy and perioperative hypotension

Characteristics	Total (*n* = 199)	NT-proBNP ≤ 450 pg/ml (*n* = 129)	NT-proBNP > 450 pg/ml (*n* = 70)	*p*
Procedural details				
Duration of intervention [IQR] - min	115 [75–163]	119 [70–181]	105 [81–154]	0.37
Duration of anaesthesia [IQR] - min	205 [152–284]	197 [149–294]	209 [155–270]	0.985
Fluid balance				
Perioperative fluid balance [IQR] - ml	950 [480–1280]	950 [480–1350]	960 [400–1220]	0.775
Amount of colloids [IQR] - ml	0 [0–500]	0 [0–500]	0 [0–500]	0.919
Amount of crystalloids [IQR] - ml	1132 [1000–1280]	1083 [1000–1656]	1227 [1000–1643]	0.675
Amount of fluid by autotransfusion [IQR] - ml	0 [0–0]	0 [0–0]	0 [0–0]	0.175
Amount of red blood cell concentrate [IQR] - ml	0 [0–0]	0 [0–0]	0 [0–0]	0.100
Amount of fresh frozen plasma [IQR] - ml	0 [0–0]	0 [0–0]	0 [0–0]	0.345
Blood loss [IQR] - ml	200 [50–400]	150 [50–400]	250 [50–500]	0.260
Urinary output [IQR] - ml	350 [20–720]	300 [0–785]	445 [150–621]	0.182
Catecholamine therapy				
Cumulative amount of norepinephrine [IQR] - μg	178 [0–554]	83 [0–436]	261 [4–784]	**0.007**
Cumulative amount of epinephrine [IQR] - μg	0 [0–0]	0 [0–0]	0 [0–0]	0.175
Perioperative Hypotension				
Incidence - no. (%)	176 (88.4)	114 (88.4)	62 (89.0)	0.966
Absolute duration [IQR] - min	14.6 [3.8–28.3]	15.7 [3.3–29.2]	13.8 [5.8–27.6]	0.786
Relative duration [IQR] - %	6.7 [1.9–13.5]	6.8 [1.9–13.6]	6.7 [2.1–12.2]	0.938
AUC [IQR] – min × mmHg	60.7 [11.5–168.6]	58.8 [10.5–167.0]	62.9 [20.2–180.4]	0.655
Relative AUC [IQR] – min × mmHg/%	25.5 [6.2–73.9]	24.4 [4.7–71.0]	28.2 [9.4–84.6]	0.624

Hypotension was defined as mean arterial pressure < 65 mmHg. Abbreviations: AUC = area under the curve

### Outcome

**Fig. 2:**
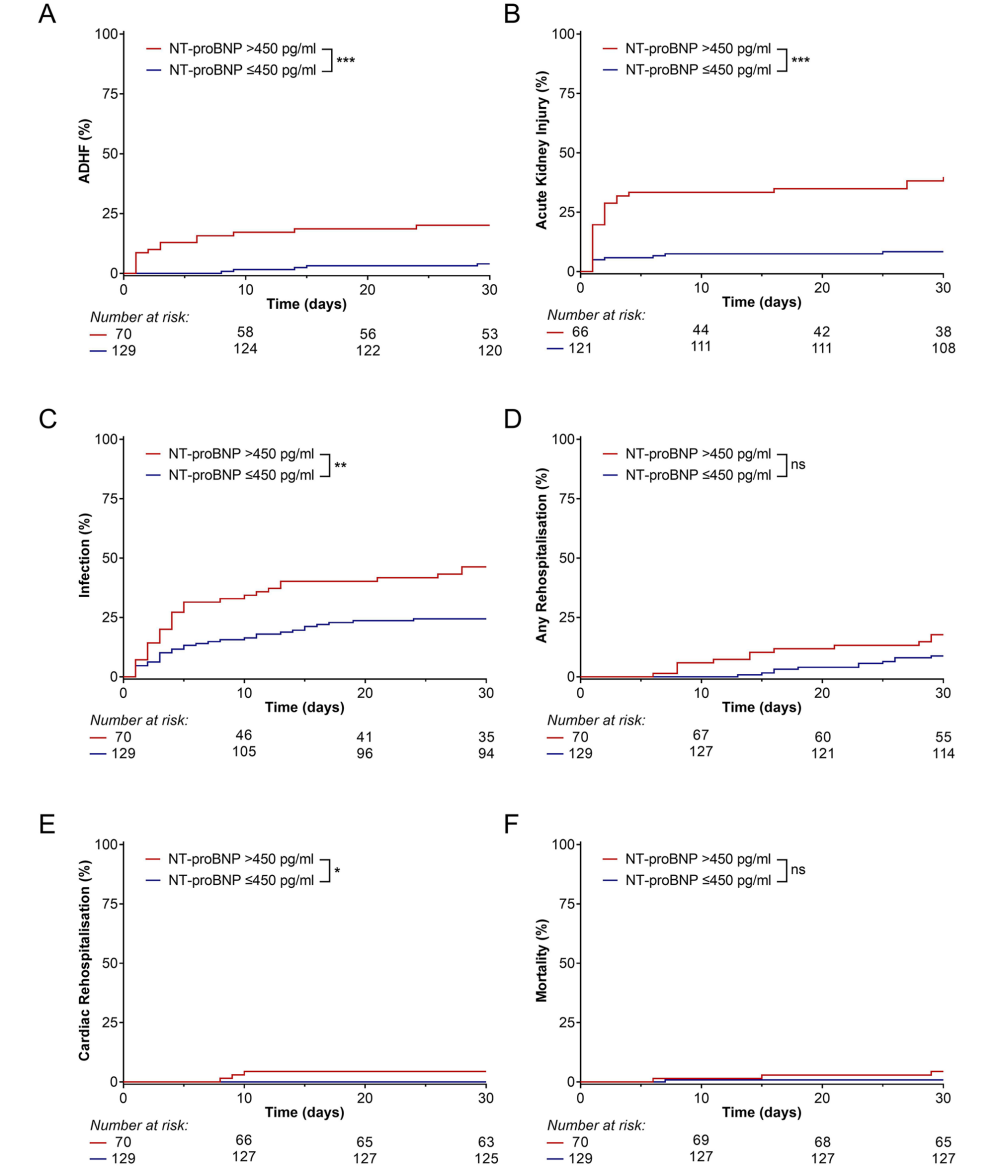
Individual components of the primary endpoint. Among the individual components of the composite morbidity endpoint, significantly higher cumulative event rates of **A** acute decompensated heart failure (ADHF), **B** acute kidney injury, and **C** any bacterial infection were observed in patients with preoperative NT-proBNP > 450 pg/ml. Although the incidence of **D** any rehospitalisation was comparable among groups, there was a higher rate of **E** cardiac rehospitalisation in patients with NT-proBNP > 450 pg/ml. However, **F** overall mortality at postoperative 30 remained comparable among groups. ***p < 0.001; **p < 0.01; *p < 0.05; n.s. = not significant

CME event rates were higher in patients with NT-proBNP > 450 pg/ml compared with NT-proBNP ≤ 450 pg/ml (70.7% vs. 32.4%, *p* < 0.001; Fig. 1C). Kaplan-Meier estimates for the exact calculated cut-off of 443 pg/ml are presented in Supplementary Fig. 1. Events occurred foremost during the first 10 PODs with great variability among the individual CME components (Fig. 2). The difference in CME rates was primarily due to significantly higher rates of ADHF (20.1% vs. 4.0%, *p* < 0.001), AKI (39.8% vs. 8.3%, *p* < 0.001), infection (46.3% vs. 24.4%, *p* = 0.001), and cardiac rehospitalisation (4.4% vs. 0.0%, *p* = 0.018) in the patients with NT-proBNP > 450 pg/ml. A significantly higher incidence of postoperative AKI was reported from all stages of AKI (Table 3). The median dose of intravenous diuretics administered was significantly higher (*p* < 0.001) and the length of intensive care unit and hospital stays were significantly longer (both *p* < 0.001) in patients with elevated NT-proBNP. Conversely, perioperative myocardial infarction (0.0% in both groups), wound healing disturbances (4.0% vs. 7.4%, *p* = 0.312), and cardiac mortality (1.5% vs. 0.0%, *p* = 0.169) were comparable between the groups. One patient with preoperative NT-proBNP > 450 pg/ml and pre-existing metastasised urothelial carcinoma died due to respiratory insufficiency caused by ADHF and pleural effusion on POD 14. All the other deaths observed in our study occurred due to non-cardiac causes, and the higher rate of any bacterial infections in patients with NT-proBNP > 450 pg/ml was mainly due to a significant higher incidence of postoperative pneumoniae (10.9% vs. 1.6%, *p* = 0. 011) and infections from other reasons (22.9% vs. 7.1%, *p* = 0. 003; Table 3).

**Table 3:** Kaplan-Meier event rates through 30-day follow-up, use of intravenous diuretics and length of stays

	Total (*n* = 199)	NT-proBNP ≤ 450 pg/ml (*n* = 129)	NT-proBNP > 450 pg/ml (*n* = 70)	*p*
Composite Morbidity Endpoint - no. (%)	90 (45.9)	41 (32.4)	49 (70.7)	**< 0.001**
Any rehospitalisation - no. (%)	23 (11.9)	11 (8.8)	12 (17.7)	0.056
Cardiac rehospitalisation - no. (%)	3 (1.6)	0 (0.0)	3 (4.4)	**0.018**
Acute decompensated heart failure - no. (%)	19 (9.7)	5 (4.0)	14 (20.1)	**< 0.001**
Acute kidney injury - no. (%)	36 (19.4)	10 (8.3)	26 (39.8)	**< 0.001**
Stage 1 - no. (%)	26 (14.4)	8 (6.7)	18 (29.8)	**< 0.001**
Stage 2 - no. (%)	5 (2.8)	1 (0.8)	4 (6.9)	**0.026**
Stage 3 - no. (%)	5 (2.8)	1 (0.9)	4 (6.3)	**0.025**
Any bacterial infection - no. (%)	63 (32.1)	31 (24.4)	32 (46.3)	**0.001**
Pneumonia - no. (%)	8 (4.6)	2 (1.6)	6 (10.9)	**0.011**
Abdominal infection - no. (%)	13 (7.1)	10 (8.1)	3 (4.9)	0.433
Urinary tract infection - no. (%)	13 (7.8)	6 (5.4)	7 (13.0)	0.089
Wound infection - no. (%)	8 (4.9)	5 (4.8)	3 (4.5)	0.717
Other - no. (%)	21 (12.4)	8 (7.1)	13 (22.9)	**0.003**
Further morbidity measures				
Myocardial infarction - no. (%)	0 (0.0)	0 (0.0)	0 (0.0)	1.0
Wound healing disturbance - no. (%)	10 (5.2)	5 (4.0)	5 (7.4)	0.312
Mortality - no. (%)	4 (2.0)	1 (0.8)	3 (4.3)	0.094
Cardiac - no. (%)	1 (0.5)	0 (0.0)	1 (1.5)	0.169
Postoperative diuretics				
Postoperative diuretics [IQR] - mg/d*	0.0 [0.0–1.4]	0.0 [0.0–0.0]	0.10 [0.0–5.9]	**< 0.001**
Length of stays				
ICU stay [IQR] - d	0.9 [0.0–1.3]	0.8 [0–1.1]	1.1 [0.8–3.5]	**< 0.001**
Hospital stay [IQR] - d	10 [6-17]	0.8 [6–13]	15 [8–21]	**< 0.001**

Abbreviations: ICU = intensive care unit; *daily furosemide equivalent dose

### Prognostic analysis

**Fig. 3:**
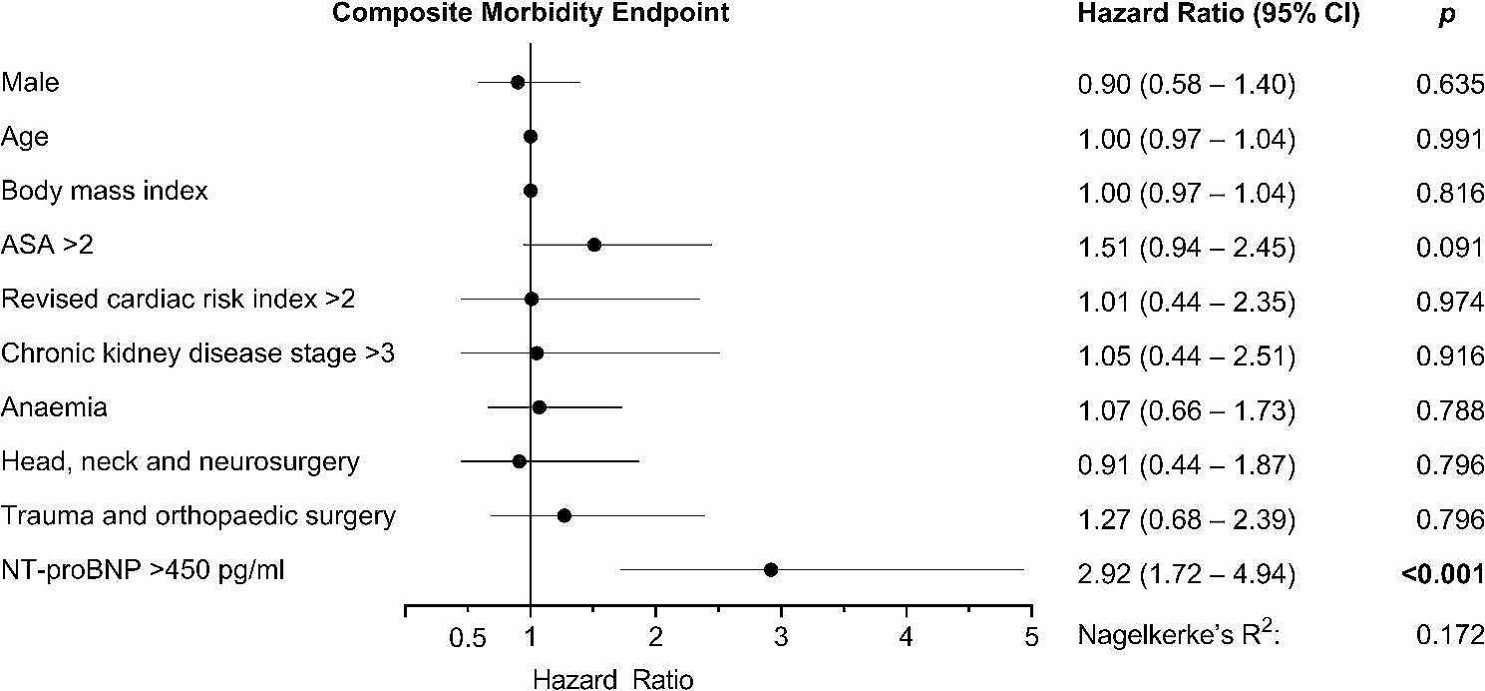
Multivariable Cox regression analysis. Forest plot hazard ratios and their respective 95% confidence intervals (CI) in multivariable Cox regression analysis for association with the composite morbidity endpoint composed of rehospitalisation, acute decompensated heart failure, acute kidney injury, and any suspected or proven bacterial infection that required treatment. ASA = American Society of Anesthesiologists

A Cox regression model was created including the NT-proBNP cut-off of 450 pg/ml and predetermined clinically relevant cut-offs which were selected a priori, including the ASA classification, the revised cardiac risk index, chronic kidney disease, and preoperative anaemia. All 199 patients with a total of 90 events were included, and the model was adjusted for sex, age, body mass index, and type of surgery. The multivariable model revealed that NT-proBNP > 450 pg/ml was an independent predictor of the CME at POD 30 (hazard ratio 2.92 [1.72–4.94], *p* < 0.001). ASA classification > 2, revised cardiac risk index > 2, chronic kidney disease stage > 3, and anaemia were unable to predict the CME (Fig. 3).

## Discussion

The findings of our monocentric observational study suggest that an NT-proBNP cut-off of 450 pg/ml can be used to estimate postoperative morbidity risk after major non-cardiac surgery with intermediate or high surgical risk in patients older than 65 years. Therefore, the calculated and clinically adjusted cut-off value, derived from point-of-care testing, is a parameter that detects patients at high risk for postoperative morbidity, which could be used in further trials to trigger distinct treatment interventions. Although postoperative mortality was comparable among the groups in our study, which is likely due to the limited sample size, short-term observation period, and the exclusion of non-elective surgeries, an overall broad study population was included. Our study showed that morbidity measures should also be considered when perioperative risk is assessed by NT-proBNP measurement, because we found a large burden of AKI, ADHF, and infections in patients with preoperative NT-proBNP > 450 pg/ml which resulted in overall prolonged ICU and hospital length of stays. Interestingly, the majority of the observed CME events occurred during the first 3 days after non-cardiac surgery.

Based on our results, it could be hypothesized that a relevant fraction of patients older than 65 years undergoing non-cardiac surgery with intermediate or high surgical risk might suffer from undiagnosed chronic HF and congestion, which could be detected with preoperative biomarker screening. However, NT-proBNP levels are known to interfere with other factors, such as weight, sex, and renal function. In general, NT-proBNP is a sensitive and specific parameter when patients are evaluated for chronic HF. Therefore, according to the current ESC guidelines for the diagnosis and treatment of acute and chronic HF, and besides the preoperative setting, chronic HF is unlikely in patients younger than 50 years with NT-proBNP < 125 pg/ml [1]. However, it must be noted that the rule-in cut-offs for chronic HF are adjusted by age, and chronic HF is likely for NT-proBNP levels ≥ 250 pg/ml in patients aged 50 to 75 years and ≥ 500 pg/ml in patients older than 75 years [21]. Although availability of preoperative echocardiography was limited in our analysis, the differences in the evaluated parameters suggest a higher prevalence of pathological findings in patients with NT-proBNP > 450 pg/ml. Furthermore, a new condition termed ‘heart stress’ has recently been introduced for patients with cardiovascular risk factors and elevated natriuretic peptides, who do not present with clinical symptoms of HF [21].

The results of our study are in line with the findings of Gualandro et al., who reported a 51% rate of de novo HF in patients with postoperative ADHF. In our cohort, more than 75% of patients with an NT-proBNP level above the calculated threshold had no prior history of HF that could be detected at the preoperative anaesthesiologic visit [14].

Interestingly, although differences in various clinical baseline characteristics were detected, NT-proBNP outweighed other parameters and risk indices, such as the ASA classification and revised cardiac risk index, in the multivariable Cox regression model. Therefore, the evaluation of preoperative NT-proBNP might enhance the predictive value of the preoperative anaesthesiologic visit. To date, established risk scores and predictive models considering surgical risk in the current guidelines have foremost focused on major adverse cardiovascular events, like cardiac death and myocardial injury or infarction [6, 12, 22]. Therefore, it is plausible why established risk estimation measures, such as the ASA classification and the revised cardiac risk index, could not predict postoperative morbidity in the multivariable model when compared to the NT-proBNP cut-off. Consequently, biomarkers like NT-proBNP could cover a wider range of outcome measures compared with clinical risk scores, which have been validated for certain specific endpoints. Nonetheless, none of the established risk scores include preoperative biomarker measurements, and no clinically applicable combination of modified risk scores, including biomarkers, is available [6]. However, evidence has emerged that biomarkers can enhance predictive models based on clinical risk scores [11, 23]. Predictive models for major adverse cardiovascular events and myocardial injury or infarction improve with the addition of NT-proBNP or high-sensitivity cardiac troponin to the revised cardiac risk score in patients undergoing major non-cardiac surgery [24, 25]. The calculated sensitivity of NT-proBNP in predicting the CME was relatively low in our study, while specificity was considerably greater when the cut-off of 450 pg/ml was chosen. Therefore, clinical risk scores with high sensitivity could prompt preoperative biomarker testing with high specificity, resulting in an overall increased predictivity of postoperative morbidity compared to either biomarker testing or a clinical risk score alone. NT-proBNP might be particularly suitable, as a high negative predictive value was observed when used as a ‘rule-out’ test [25].

It is striking that most of the events that occurred during the postoperative hospital stay occurred within the first PODs in our cohort, as most patients were still receiving comprehensive hospital care during that period. Because other studies reported similar observations, it appears that the current standard of postoperative surgical care may not fully address potential HF-related postoperative complications [11, 14, 26]. In addition, the timing of postoperative adverse events suggests that future optimised perioperative treatment protocols should begin in the preoperative setting. However, limited single preoperative interventions without dedicated peri- and postoperative adapted management strategies did not improve outcomes in previous studies. Routine preoperative echocardiography, cardiopulmonary exercise testing, or the perioperative administration of increased fractions of inspired oxygen did not improve patient outcomes [27–29]. Therefore, future efforts should focus on combining preoperative biomarker screening with peri- and postoperative treatment optimisation. In our study, patients with elevated NT-proBNP had lower Hb compared with patients with NT-proBNP below the cut-off value, suggesting that preoperative anaemia treatment should be expanded according to the current guidelines. However, despite the association between preoperative anaemia and postoperative ADHF, it could not predict our combined endpoint in the Cox regression model, which could be due to the limited sample size or selection of endpoint events [14]. Furthermore, it may be more effective to diagnose and subsequently treat preoperatively diagnosed de novo HF or optimise medications in patients with chronic congestion. Therefore, in patients with abnormalities in preoperative cardiac biomarkers, systematic cardiological evaluation, including echocardiographic assessment, should be performed in accordance with the current guidelines for the diagnosis and treatment of acute and chronic HF [1]. Herein, HF with preserved ejection fraction (HFpEF) may require particular attention, because the echocardiographic findings of our study and others suggest that HFpEF is the dominant phenotype of HF first diagnosed during the perioperative period [14].

Although all procedures were elective in our study, not every intervention, such as oncological and trauma surgery, could be postponed until newly diagnosed HF workup was complete. Therefore, optimisation strategies should include the intraoperative period. Our data suggest that routine intraoperative anaesthesiologic management of patients with elevated NT-proBNP requires the administration of higher cumulative doses of norepinephrine to maintain a sufficient mean arterial pressure to effectively prevent perioperative hypotension. Therefore, the higher incidence of AKI in patients with elevated NT-proBNP above the defined threshold could not be attributed to perioperative hypotension, although AKI is generally associated with intraoperative hypotension [30]. Potential mechanisms leading to the high incidence of AKI in our study are the well-known as complex interactions of cardiac function and renal function, which are also expressed by the higher prevalence of chronic kidney disease in patients with NT-proBNP > 450 pg/ml [31].

Fluid administration was comparable between the groups in our study. This leads us to the question whether pre-existing fluid overload - which was most likely present preoperatively, as indicated by elevated NT-proBNP as a marker of cardiac filling pressure - was further aggravated by “liberal” fluid administration. Despite these established implications in HF patients, there is an association between high fluid volumes administered during surgeries and prolonged hospital stays and an increased incidence of postoperative ileus following rectal and colon surgery [32]. Therefore, goal-directed intraoperative haemodynamic therapy, where fluid boluses are guided by haemodynamic parameters, might be beneficial in patients with elevated preoperative NT-proBNP. Goal-directed therapy algorithms guarantee that intraoperative fluid therapy is guided by cardiac stroke volume or dynamic preload parameters, such as pulse pressure variation and stroke volume variation, which predict fluid responsiveness [33]. In general, previous studies have provided evidence that goal-directed therapy can reduce the incidence of postoperative complications after major surgery [34].

Furthermore, because most events occurred in-hospital during the postoperative period, structured complication monitoring should be established during this period. Screening for signs of postoperative complications on surgical wards could reduce postoperative HF complications. HF screening can be effectively performed by specialised HF nurses, a practice that has been shown to reduce HF-related mortality and rehospitalisation in outpatient settings [35].

In summary, preoperative patient evaluation including the use of natriuretic peptide screening has the potential to identify patients at risk of multiple complications. Because single interventions have not yet convincingly demonstrated a clinical benefit in at-risk patients, a novel comprehensive interdisciplinary perioperative medical team approach, including anaesthesiologists, specialist surgeons, and cardiologists, guiding optimal treatment of the patient throughout the hospital stay, should be evaluated in future studies [6, 14].

The following limitations of our study must be acknowledged. First, the sample size was limited to 200 consecutive and elective patients undergoing surgery in general anaesthesia. Therefore, further large-scale studies with independent patient datasets are needed to validate the suggested NT-proBNP cut-off of 450 pg/ml. However, the cut-off may be suitable for use in randomised interventional trials as it can discriminate high CME event rates. Preoperative differences in echocardiographic findings between the two groups must be interpreted with caution as the overall availability of echocardiographic parameters was limited. Second, no interventions or specific perioperative protocol regulating laboratory testing at distinct time points were used due to the observational nature of the study. Therefore, clinical events may have been underestimated when clinical parameters were not measured at the same time points; in contrast, more frequent testing in one group may lead to increased detection of clinically silent events, such as first-degree AKI. There is no evidence that the latter occurred in our study because postoperative treatment was left entirely to the surgeons’ discretion, and they were unaware of the prospective biomarker study. Third, the surgical risk stratification (according to the current guidelines) we used as inclusion criteria might have been insufficient, because this risk stratification only relates to the risk of cardiovascular death, myocardial infarction, and stroke [6]. Moreover, other types of surgery that were not classified as intermediate or high risk may be relevant but were not addressed in this study. Finally, the definition of ADHF used in our study was derived from clinical parameters. Because of the observational nature of our study, patients suffering from postoperative ADHF did not undergo standardized echocardiography at this time. Therefore, left ventricular dysfunction with elevated filling pressures could not be proven, and other reasons for the occurrence of the clinical endpoint ADHF, such as hyperhydration, hypertensive pulmonary oedema, cessation of HF medication, or new-onset atrial fibrillation with tachyarrhythmia, might have also been relevant. Finally, it is possible that elevated NT-proBNP was not only a sign of cardiac dysfunction in our study but also selected pre-existing multimorbid patients within the elevated NT-proBNP cohort who were generally at a special risk for postoperative morbidity measures, such as AKI and infection.

## Conclusions

In conclusion, the findings of our monocentric observational study undergoing major non-cardiac surgery with intermediate or high surgical risk suggests the effectiveness of an easily implementable preoperative NT-proBNP cut-off of 450 pg/ml in patients older than 65 years to predict postoperative morbidity. Preoperative NT-proBNP was more effective than established risk scores, including the revised cardiac risk index and ASA classification. Further studies should focus on interdisciplinary approaches to improve postoperative morbidity through interventions in the preoperative, intraoperative, and postoperative periods.

### Electronic supplementary material

Below is the link to the electronic supplementary material.

Supplementary Material 1

## Data Availability

The datasets used and/or analysed during the current study are available from the corresponding author on reasonable request.

## Abbreviations

ADHF: Acute decompensated heart failure
AKI: Acute kidney injury
ASA: American Society of Anesthesiologists
BNP: Brain natriuretic peptide
CME: Composite morbidity endpoint
eGFR: Estimated glomerular filtration rate
ESC: European Society of Cardiology
Hb: Haemoglobin
HF: Heart failure
HFpEF: Heart failure with preserved ejection fraction
NT: ProBNP-N-terminal prohormone of brain natriuretic peptide
POD: Postoperative day
ROC: Receiver operating characteristic
