# Supplementary material for: Preoperative routine measurement of NT-proBNP predicts postoperative morbidity after non-cardiac surgery with intermediate or high surgical risk: an observational study
Source: BMC Anesthesiol. 2024 Mar 23;24:113. doi: 10.1186/s12871-024-02488-8 (PMC10960410; doi:10.1186/s12871-024-02488-8)
Supplement: Supplementary file 1 — Supplementary Material 1 [file 12871_2024_2488_MOESM1_ESM.docx]

**
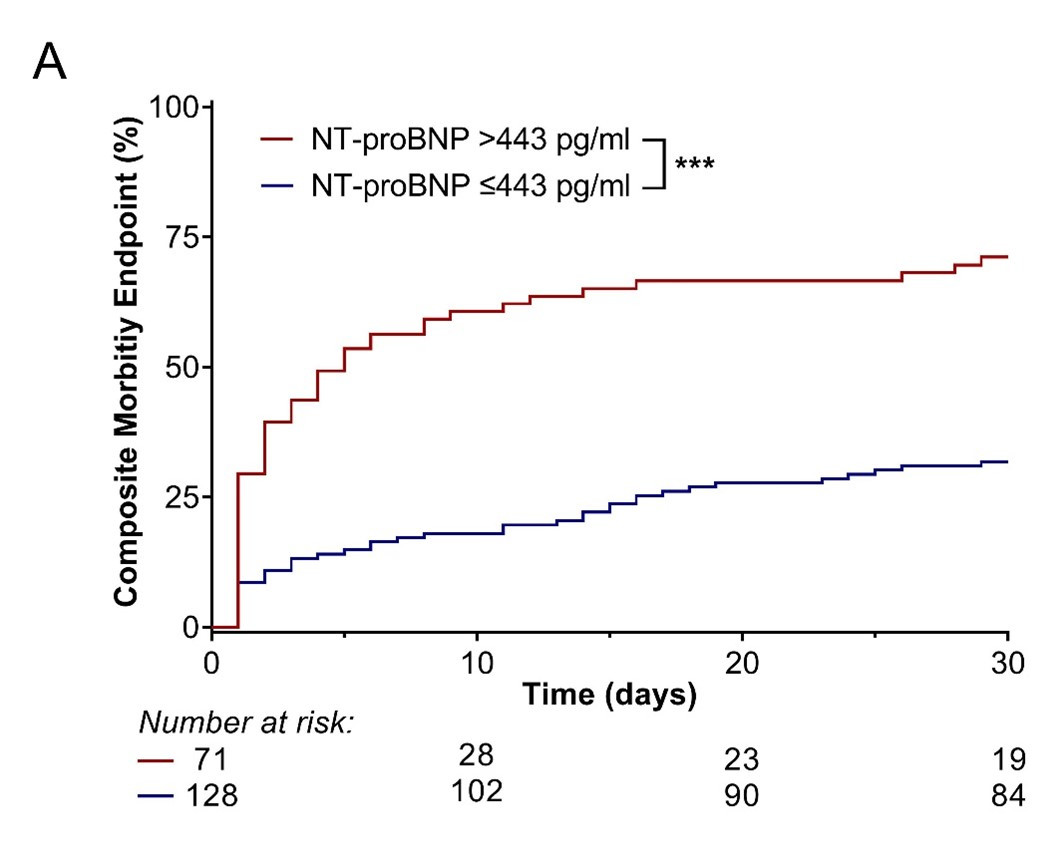
**

**Supplementary Figure 1. A** When the calculated NT-proBNP cut-off of 443 pg/ml was used to assess the risk of postoperative morbidity, composite morbidity endpoint rates were largely comparable with the cut-off of 450 pg/ml (NT-proBNP >443 pg/ml: 71.1% vs. NT-proBNP ≤443 pg/ml: 31.8%). ****p* < 0.001.
